# Assessing the Underwater Acoustics of the World's Largest Vibration Hammer (OCTA-KONG) and Its Potential Effects on the Indo-Pacific Humpbacked Dolphin (*Sousa chinensis*)

**DOI:** 10.1371/journal.pone.0110590

**Published:** 2014-10-22

**Authors:** Zhitao Wang, Yuping Wu, Guoqin Duan, Hanjiang Cao, Jianchang Liu, Kexiong Wang, Ding Wang

**Affiliations:** 1 The Key Laboratory of Aquatic Biodiversity and Conservation of the Chinese Academy of Sciences, Institute of Hydrobiology, Chinese Academy of Sciences, Wuhan, P. R. China; 2 University of Chinese Academy of Sciences, Beijing, P. R. China; 3 School of Marine Sciences, Sun Yat-sen University, Guangzhou, P. R. China; 4 Hongkong-Zhuhai-Macao Bridge Authority, Guangzhou, P. R. China; 5 Transport Planning and Research Institute, Ministry of Transport, Beijing, P. R. China; Virginia Commonwealth Univ, United States of America

## Abstract

Anthropogenic noise in aquatic environments is a worldwide concern due to its potential adverse effects on the environment and aquatic life. The Hongkong-Zhuhai-Macao Bridge is currently under construction in the Pearl River Estuary, a hot spot for the Indo-Pacific humpbacked dolphin (*Sousa chinensis*) in China. The OCTA-KONG, the world's largest vibration hammer, is being used during this construction project to drive or extract steel shell piles 22 m in diameter. This activity poses a substantial threat to marine mammals, and an environmental assessment is critically needed. The underwater acoustic properties of the OCTA-KONG were analyzed, and the potential impacts of the underwater acoustic energy on *Sousa*, including auditory masking and physiological impacts, were assessed. The fundamental frequency of the OCTA-KONG vibration ranged from 15 Hz to 16 Hz, and the noise increments were below 20 kHz, with a dominant frequency and energy below 10 kHz. The resulting sounds are most likely detectable by *Sousa* over distances of up to 3.5 km from the source. Although *Sousa* clicks do not appear to be adversely affected, *Sousa* whistles are susceptible to auditory masking, which may negatively impact this species' social life. Therefore, a safety zone with a radius of 500 m is proposed. Although the zero-to-peak source level (SL) of the OCTA-KONG was lower than the physiological damage level, the maximum root-mean-square SL exceeded the cetacean safety exposure level on several occasions. Moreover, the majority of the unweighted cumulative source sound exposure levels (SSELs) and the cetacean auditory weighted cumulative SSELs exceeded the acoustic threshold levels for the onset of temporary threshold shift, a type of potentially recoverable auditory damage resulting from prolonged sound exposure. These findings may aid in the identification and design of appropriate mitigation methods, such as the use of air bubble curtains, “soft start” and “power down” techniques.

## Introduction

Over the past few decades, anthropogenic (human-generated) noise in aquatic environments has generated worldwide concern due to its potential adverse effects on the environment and aquatic life [1]–[5]. Of particular concern are the intense impulsive sounds from explosive detonations, seismic surveys and pile driving, common activities in the construction of renewable-energy marine wind farms, docks and bridges. The effects on marine mammals have been of particular interest [6]–[9]. This concern is partly due to the protected status of marine mammals under state laws and international conventions, such as the Convention on International Trade in Endangered Species of Wild Fauna and Flora, as well as their vulnerability to ambient noise. Cetaceans have a sophisticated acoustic sensory system with wideband hearing sensitivity [10], and they are heavily dependent on the acoustic environment for many life functions. They have evolved sophisticated vocalizations and multiple sound-reception pathways, and they rely on acoustic stimuli for social interaction, navigation and foraging in the marine environment [10].

**Figure 1:**
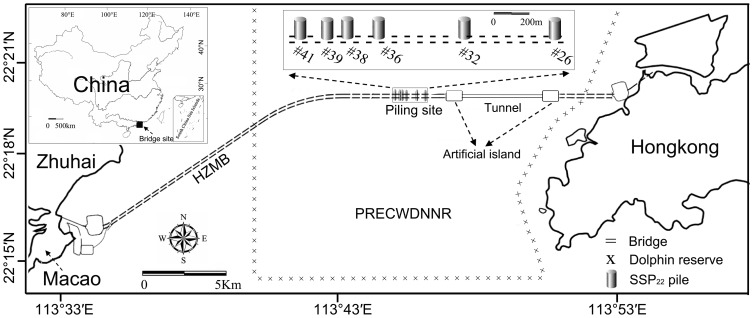
Map of the OCTA-KONG vibration monitoring area. HZMB: Hongkong-Zhuhai-Macao Bridge; PRECWDNNR: The Pearl River Estuary Chinese White Dolphin National Nature Reserve. The eastern boundary of the PRECWDNNR is also the boundary of the Zhuhai and Hongkong Special Administrative Regions. An exclusion zone of 200 m radius was established along the bridge.

The Greater Pearl River Delta is one of the most economically developed regions in China [11]. However, land transport between its western (such as the Zhuhai and Macao Special Administrative Regions) and eastern regions (such as the Hongkong Special Administrative Region) is limited by the Pearl River Estuary (PRE). To increase the region's economic competitiveness and to facilitate economic collaboration, e.g., by reducing the costs involved in transporting people and goods between the regions, the Hongkong-Zhuhai-Macao Bridge (HZMB) is being constructed to connect these three cities. The HZMB Island Tunnel Project is a large-scale, cross-boundary sea crossing involving more than 300 supporting bridge piles, an underwater tunnel and two artificial islands (Fig. 1). Construction began on 15 December 2009 and is expected to continue into 2016 [12].

The PRE (22°16′S; 113°43′E) is a hot spot for the Indo-Pacific humpbacked dolphin (*Sousa chinensis*, locally called the Chinese white dolphin), which is distributed in shallow coastal waters from South Africa in the west to southern China in the east [13]. This species is currently assessed as Near Threatened; the *chinensis*-type geographic form (found from the east coast of India to China) is categorized as Vulnerable by the International Union for the Conservation of Nature Red List of Threatened Species [13] and as a Grade One National Key Protected Animal by China's Wild Animal Protection Law, issued in 1988. The population size of humpbacked dolphins in the PRE was estimated to be 2555 and 2517 during the wet and dry seasons, respectively [14], representing the largest known humpbacked dolphin population in China [15], [16] and the world [14], [17]. To better protect the dolphin population, the Pearl River Estuary Chinese White Dolphin National Nature Reserve (PRECWDNNR) (Fig. 1) was established in the PRE in 1999.

Unfortunately, the HZMB, which uses thousands of piles driven into the bottom of the estuary, is located across the PRECWDNNR. To minimize any adverse effects on protected species, the following strategies were adopted: (1) An underwater tunnel was designated to replace a bridge structure in the core area of the reserve (Fig. 1). (2) A marine mammal safety zone (an exclusion zone of 200 m radius [18]) was established in the vicinity of the bridge construction sites. Qualified marine mammal observers scan for the presence of marine mammals within the exclusion zone. If marine mammals are observed in the safety zone, operations halt until the animals have left the zone. (3) An acoustic deterrent device (Future Oceans 70 kHz Dolphin Pinger; Future Oceans, Queensland, Australia) that emits a 145 dB signal for 300 m every 4 s is used to warn any marine mammals away from the safety zone both before and during construction. (4) A hydraulic vibration hammer is used for pile driving in addition to an impact hammer, which generates substantially louder impulse sounds.

However, due to a limited understanding of the sound produced by the construction activities, the safety zone was not established based on robust experimental or theoretical information. Efforts to protect animals are generally hampered when only limited data are available for establishing criteria for interim protection. Research on the characteristics of the underwater sound field produced during bridge construction is needed. In particular, pile driving, which produces loud underwater sounds, requires study to improve environmental impact analyses and aid in the identification and design of appropriate mitigation methods [19].

Compared with the conventionally used impact hammers, the vibratory hammer is a much more economical tool for construction companies [20]. In addition to its ability to extract piles, other advantages include (1) a lighter weight than conventional hammers, (2) faster operation at a lower noise level than conventional hammers and (3) lack of requirement of a temporary guide frame for driving free-standing piles [20]. Accordingly, it represents an alternative tool, or a complementary tool, to impact hammers from a conservation perspective.

In the waters of western Hong Kong, *S. chinensis* has been observed to travel at higher speeds during percussive pile driving. Moreover, the animals tend to partially and temporarily abandon the pile driving area [21]. Given that the peak pressure levels produced by a normal vibration hammer during the driving of normal, cast-in-steel-shell piles range from approximately 175 dB to 205 dB [19], the OCTA-KONG (American Piledriving Equipment Inc., Kent, WA, USA), which is the world's largest vibration hammer and is capable of driving and extracting piles, may impose a substantial threat to marine mammals. The use of this hammer further emphasizes the need for an assessment of underwater noise in and around the HZMB.

The present study had two main purposes. The first was to characterize the acoustic properties of the operating sounds of the OCTA-KONG, including pile driving and extraction. The second was to assess the potential impacts of this anthropogenic noise on *Sousa* with respect to three factors: *Sousa* sounds (whistles and clicks) recorded in the same district during a previous dolphin acoustic survey by the first author;*Sousa* audiograms [22], [23]; the safety exposure level established by the U.S. National Marine Fisheries Service (NMFS) [24] and the marine mammal noise exposure criteria proposed by a panel of experts from a wide range of disciplines in acoustic research [7] and the National Oceanic and Atmospheric Administration (NOAA)[25].

## Methods

### Vibration piling

**Figure 2:**
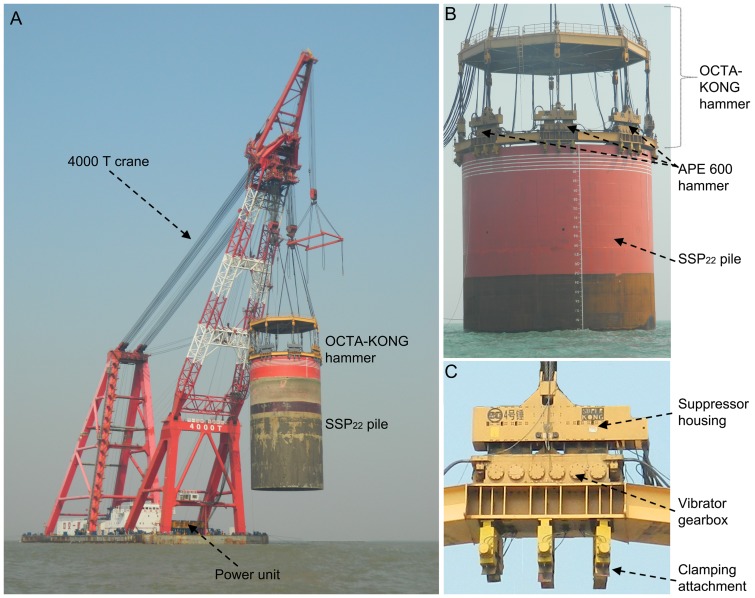
OCTA-KONG vibration operation. During vibration, the pile and hammer are rigidly connected (A). The OCTA-KONG was a tandem combination of 8× APE 600 (B), with each APE600 composed of a suppressor housing, a vibrator gearbox and a clamping attachment (C).

The OCTA-KONG is the world's largest hydraulic vibratory driver/extractor. It consists of a Multiple Linked Vibro System with 8 APE 600's connected in a tandem combination (Table 1, Fig. 2), with each APE 600 powered by a Model 1200 power unit (Fig. 2). The OCTA-KONG was used to drive 22 m diameter steel shell piles (SSP_22_) during the construction of the main wall of the two artificial islands (Fig. 1) from 15 May 2011 to 25 December 2011, and more than 120 SSP_22_ were installed. It was subsequently used to drive and/or pull SSP_22_ at the construction sites of the bridge piers from #16 to #53 and from #60 to #89 beginning 15 October 2012; the estimated completion date is in June 2015 (Zeng TQ, personal communication).

**Table 1:** Specification of the OCTA-KONG hammer, power unit and SSP_22_ pile.

Hammer		Power unit		Pile	
Type	OCTA-KONG	Type	CAT32	Type	Steel shell pile
Total Drive Force	40000 000 N	Maximum power	882 600 W(1200 HP)	Diameter	22 m
Frequency	6.67 Hz–23.33 Hz (400–1400 vpm)	Operating speed	800 r/min to 2050 r/min(rpm)	Pile wall width	0.016 m
Pile clamp force	1176 000 N*	Maximum drive pressure	33 096 Pa	Height	39 m–60 m
Line pull for extraction	3131 000 N*	Clamp pressure	33 096 Pa	Weight	450 000 kg–600 000 kg

OCTA-KONG: a Multiple Linked Vibro System with 8× APE 600's connected in a tandem combination; vpm  =  vibrations per minute; rpm  =  revolutions per minute; hp  =  horsepower. * indicates the results for each APE 600 hammer. The rpm of the hammer was controlled by the vpm of the power unit and was approximately vpm/1.44.

#### Vibration piling components

The major components of the hammer are as follows: (1) the suppressor housing (bias-weight), with a rubber elastomer isolated suppressor; (2) the vibrator gearbox, incorporating the phased high-amplitude eccentric weights; and (3) the clamping attachment (Fig. 2C).

#### Principles of vibration piling

During pile driving and extraction, the pile and hammer (except for the suppressor housing) are rigidly connected by the clamping attachment, forming a hammer-pile complex oscillating exciter (Fig. 2). Vibration is caused by the vertical movement produced by the centrifugal force that arises when the pairs of eccentrics are counter-rotated [20].The continuous pulses of energy transferred from the hammer-pile complex to the soil can temporarily change the stress-strain behavior, such as soil displacement (e.g., at the penetrating pile tip), and they can create excess pore water pressures or even complete fluidization of the soil. As a consequence, the frictional (i.e., both the internal friction of the soil and the pile-soil friction) and tip resistances are strongly reduced during vibratory driving, enabling the pile to penetrate under the low vertical thrust produced from the combined action of centrifugal force and the self-weight of the hammer-pile complex [20]. The detailed mechanics are not discussed further here.

#### Piling procedure

The SSP_22_ were 38 m–60 m long and weighed 450 000 kg–600 000 kg (Table 1). During the initial stage of pile installation (i.e., the pre-OCTA-KONG driving session), one of the SSP_22_ was rigidly connected to the clamping attachment of the OCTA-KONG, moved to the predesignated location by crane (Fig. 2) and sunk approximately 20 m by the self-weight of the hammer-pile complex. During the OCTA-KONG driving session, the hammer was used to further drive down the hammer-pile complex to the desired depth. The average sink depth during OCTA-KONG piling was 5 m (range 4 m to 6 m), depending on the substrate. During pile extraction, the OCTA-KONG was powered at the outset to reduce the pile-soil friction and to extract the pile using the line pull of the crane. At a certain point, the operation of the OCTA-KONG was stopped, and only the crane was used to extract the pile.

### Ethical statement

Permission to conduct the study was granted by the Ministry of Science and Technology of the People's Republic of China. The research permit was issued to the Institute of Hydrobiology of the Chinese Academy of Sciences (Permit number: 2011BAG07B05).

### Acoustic data recording system

Two sets of recording systems were adapted for underwater sound recording. The first was a boat-based system (hereafter referred to as BS) consisting of a Reson piezoelectric hydrophone (model TC-4013-1; Reson Inc., Slangerup, Denmark), a 1 MHz bandwidth EC6081 voltage pre-amplifier with a band-pass filter (model VP2000; Reson Inc.), a high-speed, 16 bit, multifunction data acquisition (DAQ) card (model NI USB-6251 BNC; National Instruments (NI), Austin, TX, USA),a laptop computer and LabVIEW 2011 SP1 (NI) software. Underwater signals were detected with a Reson hydrophone (sensitivity: −211 dB re 1 V/µPa at 1 m distance; frequency response: 1 Hz to 170 kHz +1/−7 dB) and conditioned by a VP2000 pre-amplifier. Further high-pass filtering at 10 Hz was conducted to reduce system and flow noise, and low-pass filtering at 250 kHz was conducted to prevent aliasing before inflow into the NI USB-6251 BNC DAQ card. The acoustic data were then stored directly on the hard drive of a computer in binary format with a sampling rate of 512 kHz, using LabVIEW software. The second recording system was a Song Meter Marine Recorder (hereafter referred to as SM2M), which included an HTI piezoelectric hydrophone (model HTI-96-MIN; High Tech, Inc., Long Beach, MS, USA) with a sensitivity of −165 dB re 1 V/µPa at 1 m distance and a frequency response of 2 Hz–48 kHz +/−2 dB. It also included a programmable autonomous signal processing unit, integrated with a band-pass filter and a pre-amplifier, which can log data at a resolution of 16 bits and up to a 96 kHz sample rate, with a storage capacity of 512 GB (4×128GB SDXC cards). The signal processing unit was sealed inside a waterproof PVC housing and was submersible to a depth of 150 m. The Reson hydrophone and the SM2M system were calibrated prior to shipment from the factory. The remaining components of the BS system, including the amplifier, filter, DAQ card, LabVIEW software and laptops, were lab-calibrated prior to the field survey by inputting a calibration signal generated by an OKI underwater sound level meter (model SW1020; OKI Electric Industry Co., LTD., Tokyo, Japan). Signal transmission was also simultaneously monitored with an oscilloscope (model TDS1002C; Tektronix Inc., Beaverton, OR, USA).

### Data collection

Acoustic recordings were made on 5 days between 21 October, 2013 and 4 January, 2014 at the construction site of the HZMB, China (21°16′–21°16′S; 113°33′–113°55′E) (Fig. 1, Table 2). Surveys were conducted from a 7.5 m recreational power boat with a 102 970 W (140 horsepower) outboard engine. Both stationary and floating recording methods were used during sound recording. For stationary recording, either peripheral static buoys were used to suspend the submersible SM2M or the research vessel was moored with an anchor to form a static platform for the boat-based BS recording system. For floating recordings, the vessel's engine was turned off after approaching a pile, allowing the boat to drift. The recording system was then deployed from the side of the boat. If the boat drifted too far from the pile, recording was stopped, and the boat was repositioned. During sound recording, the vessel's engine remained off. The hydrophones were deployed to 2 m depth using an attached weight to limit movement due to water flow. Furthermore, pile driving was performed primarily during the slack water period, when tidal influence on the water depth and currents were both minimal.

**Table 2:** Descriptions of OCTA-KONG vibration sites, sound recording equipment and method.

	Type	Site	Longitude	Latitude	System	Recording type	Depth(m)	Duration(s)
10/21/2013	Piling	#32	22°16′59″	113°45′40″	SM2M	Fixed	8	137
12/4/2013	Piling	#39	22°16′60″	113°45′25″	BS	Float	7	150
12/13/2013	Piling	#38	22°16′61″	113°45′17″	BS	Fixed	8	142
12/23/2013	Piling	#41	22°16′62″	113°45′05″	BS	Fixed	7	156
1/4/2014	Piling	#36	22°16′63″	113°45′25″	BS	Fixed	7	139
12/23/2013	Extract	#26	22°16′64″	113°46′03″	BS, SM2M	Fixed and float	8	2218

Duration: the OCTA-KONG vibration duration.

The distance to the construction site was measured using Nikon laser rangefinders (model Ruihao 1200S; Nikon Imaging (China) Sales Co., Ltd., Shanghai, China) with a performance range of 10 m to 1100 m and an accuracy of ±1 m. The locations for both stationary recording and floating recording were also logged using a GPS receiver (model GPSMAP 60CSx; Garmin Corporation, Sijhih, Taiwan). The water depth and quality, including temperature, salinity and pH, was measured with a Horiba Multi-parameter Water Quality Monitoring System (model W-22XD; Horiba, Ltd., Kyoto, Japan). Ambient noise was recorded before OCTA-KONG piling operations.

### Acoustic data analysis

Acoustic signals, including OCTA-KONG vibration sounds and ambient noise, were continuously sliced into a time window segment of 1 s. Segments with obvious interference were deleted. Analysis was conducted with SpectraLAB 4.32.17 software (Sound Technology Inc., Campbell, CA, USA) and MATLAB 7.11.0 (The Mathworks, Natick, MA, USA) routines and custom programs.

#### Sound pressure levels (*SPLs*) and sound exposure levels (*SELs*)

The measured parameters included sound pressure levels (SPLs) and sound exposure levels (SELs). *SPLs* were derived directly from the pressure metrics, including the zero-to-peak sound pressure (i.e., the maximum of the unweighted absolute instantaneous sound pressure in the measurement bandwidth (*p_max_*)) and the root-mean-square sound pressure (i.e., the average of the square of the unweighted instantaneous sound pressure (*p(t)*) in the measurement bandwidth integrated over the analyzed signal duration (*T*)). The zero-to-peak SPL (*SPL_zp_*) is ten times the logarithm to the base 10 of the ratio of the square of the zero-to-peak sound pressure to the square of the reference sound pressure of 1 µPa (*p_ref1_*). Similarly, the root-mean-square SPL (*SPL_rms_*) is ten times the logarithm to the base 10 of the ratio of the square of the root-mean-square sound pressure to the square of the reference sound pressure of 1 µPa. The single SEL (*SEL_ss_*) is ten times the logarithm to the base 10 of the ratio of the integral of the squared sound exposure of a signal of 1 s time window to the reference sound exposure of 1 µPa^2^s (*p_ref2_*). Absolute pressure levels were derived by subtracting the sensitivity of the hydrophone and the gain due to the amplifier [10].

Spectrogram, power spectral density and 1/3 octave band frequency spectrum

The frequency composition of the signals was determined using spectrograms, which express a signal's amplitude, frequency and time, portraying amplitude as a graph plotted in a dark color on a two-dimensional time-frequency plane [10]. Power spectral density (PSD) level routines (dB re 1 µPa^2^ Hz^−1^), i.e., narrowband spectra in 1 Hz bands, which represent the averaged sound power in each 1 Hz band, were applied to investigate detailed tonal signatures [26]. The 1/3 octave band frequency spectrum, i.e., the sum of the squared pressure of all 1 Hz bands within a 1/3 octave, was investigated to assess impacts on mammalian hearing, as 1/3 of an octave approximates the effective filter bandwidth of cetaceans [5]. Both the spectrograms and narrowband spectra were obtained using the fast Fourier transform (FFT) method, combining a Hanning smoothing window function with an overlap of 85% for the averaging. For the BS (sample rate 512 kHz) and SM2M data (sample rate 96 kHz), the FFT size was 262 144 samples and 65 536 samples, respectively, resulting in a frequency grid resolution of 1.95 Hz and 1.46 Hz, respectively, and a temporal grid spacing of 76.80 ms and 102.40 ms, respectively. Narrowband spectra were further normalized to PSD by dividing by the frequency grid resolution.

#### Cetacean auditory weighted SEL (*SELws*)

As the damage risk criteria for marine mammals exposed to noise should incorporate the exposure frequency [27], cetacean auditory weighting (CA-weighting) [25] functions were used to incorporate the animals' auditory sensitivity to certain frequencies by emphasizing those frequencies where sensitivity to noise is high and de-emphasizing frequencies where sensitivity is low. The CA-weighting function (*W_CA_(f)*) was merged with a marine mammal weighting function (*W_M_(f)*, Equation 1) [7] and an equal-loudness weighting function curve (*W_EQL_(f)*, Equation 2). Function *W_EQL_(f)* was derived from bottlenose dolphin (*Tursiops truncatus*) frequency-specific temporary threshold shift data [27], [28] and equal-loudness contours [29]). Equal-loudness contours represent the SPLs of a sound that are perceived as equal in loudness magnitude in a testee as a function of sound frequency. They are considered to reveal the frequency characteristics of the testee's auditory system [30]. The contours are derived from subjective loudness experiments that ask candidates to judge the relative loudness of two tones of different frequencies [29], [31]. At each frequency, the amplitude of the *W_CA_(f)* is defined using the larger value from the two component curves (Equation 3). The cetacean auditory-weighted SEL (*SEL_ws_*) is ten times the logarithm to the base 10 of the ratio of the integral of the squared sound exposure of an CA-weighted signal of 1 s time window to the reference sound exposure of 1 µPa^2^s. This *SEL_ws_* can be simplified (Equation 4), as the integral of the squared sound exposure of an CA-weighted signal is equal to the overall energy of the CA-weighted PSD contour (*PSD_W_(f)*) multiplying its frequency resolution.
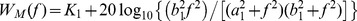
(1)




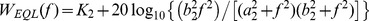
(2)




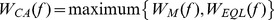
(3)




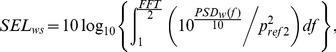
(4)where *W(f)* is the weighting function amplitude (in dB) at frequency *f* (in Hz), *a* and *b* are constants related to the lower and upper hearing limits (the “roll off” and “cut off” frequencies), respectively, and *K* is a constant used to normalize the equation at a particular frequency [32]. For *Sousa*, which belongs to the mid-frequency cetacean functional hearing group, *K_1_*, *a_1_* and *b_1_* are −16.5, 150 and 160 000, respectively, and *K_2_*_,_ *a_2_* and *b_2_* are 1.4, 7829 and 95 520, respectively [25], [32].

#### Source levels and source SELs

OCTA-KONG source levels (SLs), including the zero-to-peak SL (*SL_zp_*, dB re 1 µPa), root-mean-square SL (*SL_rms_*, dB re 1 µPa), and source SELs (SSELs), including unweighted SSEL (*SSEL_ss_*, dB re 1 µPa^2^s) and CA-weighted SSEL (*SSEL_ws_*, dB re 1 µPa^2^s), were obtained by combining measures of received level (*RL*) and transmission loss (*TL*) (Equation 5). *TL* was estimated from the distance from the source (*r*) as a result of the depth-dependent spreading loss plus frequency-dependent absorption (Equation 6) [33]. 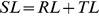
(5)




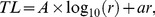
(6)where *r* is the range in meters; *A* is the spreading loss coefficient, which generally varies from 10 (cylindrical spreading) to 20 (spherical spreading); and *a* is the frequency-dependent absorption coefficient in dB/m. As the dominant frequency of vibration pile driving was below 10 kHz [19], the absorption term does not significantly contribute to transmission loss and can generally be ignored for those recordings with greatest measurement ranges of less than 1 km [34], [35]. Therefore, Equation 6 can be simplified to Equation 7 for the estimates of *SL_zp_, SL_rms_, SSEL_ss_* and *SSEL_ws_*. Sound propagation in shallow water environments (<200 m deep) is complex [33]. Attenuation may vary with depth depending on the sediment type, pressure and sediment porosity [36], and the frequency dependence of the acoustic response is sensitive to the details of the geoacoustic structure of the seabed [37]. Previous geophysical studies indicated that the surficial sediments of the bridge construction site were almost flat (TQ Zeng, personal communication). Cores taken in the vicinity of the bridge construction site indicated that the sediment was largely Quaternary sediment with approximately five layers. The top layer, deposited during the Holocene series, consists primarily of silt-clays. The second to the fourth layers, deposited during the Pleistocene series, are predominantly sand, gravel and clay. Mudstone occurs at a depth of approximately 70 m below the sea floor. The fifth layer consists of Yansanian granites (TQ Zeng, personal communication).The transmission loss equation was derived by fitting a least squares regression to the *SPL_zp_, SPL_rms_, SEL_ss_* and *SEL_ws_* measured at different distances during pile driving and extraction using the floating recording method. The derived equation was also used to estimate the source level of other piling sites where the stationary recording strategy was adopted.
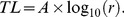
(7)


#### Unweighted and CA-weighted cumulative SSEL

Cumulative SSEL is ten times the logarithm to the base 10 of the ratio of the summation over a specified duration of sound exposures to the reference sound exposure of 1 µPa^2^s. It can be simplified as the average *SSEL_ss_* for the unweighted cumulative SSEL (*SSEL_cum_*, Equation 8) and as the average *SSEL_ws_* for the CA-weighted cumulative SSEL (*SSEL_wcum_*, Equation 9) plus the log transformation of the duration of sound exposure divided by the duration of the 1 s reference time window (*t_ref_*).
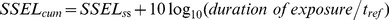
(8)




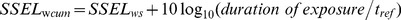
(9)


#### Audibility range

Sound audibility is determined by both external conditions, such as the characteristics of received sound and background noise conditions, and internal conditions, such as the hearing capability of the receiving system (also called the hearing audiogram). As the lowest frequency of the available *Sousa* audiogram was 5.6 kHz [22], [23], we were unable to analyze sound audibility for low-frequency sound by referencing the audiogram. Thus, the audible sound range was conservatively estimated as the range from which the sound source is attenuated by absorption and spreading loss with distance, measured at the point where the received sound is equal to the ambient noise level. The sound audible range was estimated using the transmission loss, Equation 9, to incorporate the deviation between the SL of the OCTA-KONG and the ambient noise level (the spreading loss coefficient derived above was adopted here).

#### Possible impacts on *Sousa*

The potential effects of anthropogenic noise on marine mammals include, but are not limited to, behavioral responses, auditory masking, and physiological effects [5]. Potential behavioral responses include exposure avoidance, behavioral disturbance or no response [7]. Auditory masking refers to the disruption of the reception of auditory signals by noise in the adjacent frequency bands (the so-called critical band) [7], resulting in partial or complete reduction in the audibility of the signals [7], [38], [39]. Physiological effects include temporary or (in extreme cases) permanent threshold shifts (TTS, PTS), a type of increase in the threshold of the audibility portion of an individual's hearing range or at a specified frequency above a previously established reference level producing states of temporary and recoverable shifts (TTS) or permanent, irreversible ones (PTS) [7]. As no *Sousa* were encountered during the recording period, documenting the behavioral responses was beyond the scope of the present study. Previous recordings of *Sousa* acoustics, including dolphin clicks and whistles recorded from within the same district, and *Sousa* audiograms [22], [23] were used to analyze potential auditory masking. The *Sousa* whistles and clicks were recorded by following a focal group of dolphins (an aggregation of dolphins that were engaged in the same behavior and separated by less than 100 m) [40]. Using the vocalizations to determine the animals' location was difficult because only one hydrophone system was used. However, we can confirm that the recorded dolphin sound was from the focal group because no other groups of dolphins were present within approximately 1000 m. During the sound recording, the location of the dolphins was determined within a 50 m radius of our boat based on the successive sites at which they were observed to surface and breathe. The OCTA-KONG sound level was compared with both the cetacean safety exposure level and the proposed acoustic threshold levels for the onset of TTS and PTS for the analysis of potential physiological effects. The cetacean safety exposure level established by NMFS is 180 dB (*SPL_rms_*) [24], and the proposed PTS and TTS acoustic threshold levels for *Sousa* (which are mid-frequency cetaceans) exposed to vibration driving noise (a non-impulsive sound source) are: (1) *SL_zp_* of 230 dB and 224 dB re 1 µPa, respectively; (2) *SSEL_cum_* of 195 dB and 215 dB re1 µPa^2^s, respectively; and (3) *SSEL_wcum_* of 178 dB and 198 dB re 1 µPa^2^s, respectively [25], using whichever level is first exceeded.

### Statistical analysis

Statistical analyses were conducted using SPSS 16.0 (SPSS Inc., Chicago, IL, USA). Descriptive statistics of all measured SPLs (*SPL_zp_* and *SPL_rms_*) and SELs (*SEL_ss_* and *SEL_ws_*) were obtained, including means, standard deviations (SD) and ranges (minimum - maximum values). The mean SPLs and SELs were calculated in Pa and converted to dB. A Levene's test and a Kolmogorov-Smirnov test were used to analyze the homogeneity of the variances and data normality, respectively. Nonparametric methods [41] were adopted for parameters that were non-normally distributed (Kolmogorov-Smirnov test: *p*<0.05). A Mann-Whitney U-test [41] was applied to analyze whether the SPLs and SELs of the OCTA-KONG varied significantly between pile driving and pile extraction (by comparing data recorded at the same distance to the pile and using the same system) and to test for differences in recorded noise level between the two recording systems. A Kruskal–Wallis test [41] was adopted to examine the overall ambient noise differences across different recording days. A Duncan's multiple comparison test [41] was used for post hoc comparisons of differences in ambient noise level among different recording days. Differences in the ambient noise level among different times within the same day were tested using a Mann-Whitney U-test. Differences were considered significant at *p*<0.05.

## Results

**Figure 3:**
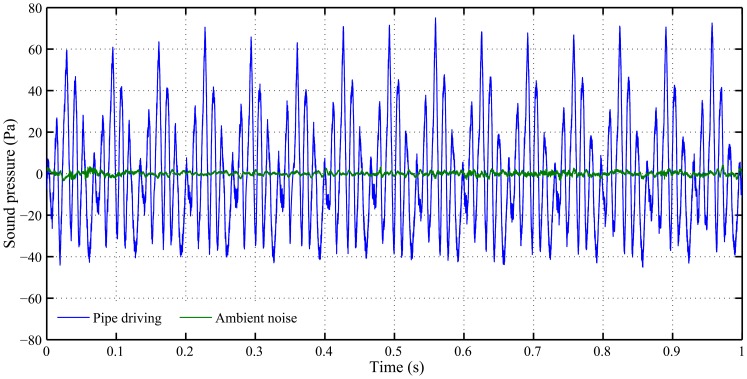
Wave form of the OCTA-KONG SSP_22_ #32 vibration sound and ambient noise. The fundamental frequency of the vibration sound was 15 Hz.

OCTA-KONG pile driving (Fig. 3) was recorded on 5 days at the sites between SSP_22_ #32 and #41, and pile extraction was monitored on SSP_22_ #26 (Table 2). One floating recording for both piling and extraction was obtained (Table 2). Water depths at the recording sites were shallow, ranging from 7 to 8 m (Table 2).

### SPL_zp_, SPL_rms_, SEL_ss_ and SEL_ws_

The acoustic signals were sliced into a time window of 1 s segments; therefore, *SPL_rms_* is numerically equivalent to *SEL_ss_*. Over all of the recording sessions, *SPL_zp_* ranged from 146.99 dB to 164.49 dB and 140.83 dB to 164 dB for pile driving and extraction, respectively (Table 3). Both the *SPL_rms_* and *SSEL_ss_* ranged from 137.77 dB to 153.11 dB and 128.83 dB to 154.58 dB for pile driving and extraction, respectively (Table 3).The OCTA-KONG vibration noise recorded by the BS system at a distance of 70 m during the driving of SSP_22_ #41 and the extraction of SSP_22_ #26 was not significantly different in *SPL_zp_* (Mann-Whitney U-test; z = −1.21, df = 337, *p* = 0.23) but significantly different in *SPL_rms_, SEL_ss_* and *SEL_ws_* (Mann-Whitney U-test: z = −9.03, df = 337, *p*<0.01;Mann-Whitney U-test: z = −9.03, df = 337, *p*<0.01 and Mann-Whitney U-test: z = −14.05, df = 337, *p*<0.01; respectively) (Table 3). Ambient noise was inspected aurally and via spectrogram, and no bio-acoustic sound generation was observed. The ambient noise could have resulted primarily from wind-driven waves and sea-surface agitation [42]. No significant differences in *SPL_zp_*, *SPL_rms_*, *SEL_ss_* and *SEL_ws_* were observed between the ambient noise recorded by the BS and SM2M systems at SSP_22_ #26 (Mann-Whitney U-test: z = −0.30, df = 125, *p* = 0.76; Mann-Whitney U-test: z = −1.73, df = 125, *p* = 0.08; Mann-Whitney U-test: z = −1.73, df = 125, *p* = 0.08 and Mann-Whitney U-test: z = −1.35, df = 125, *p* = 0.18; two-tailed; respectively) (Table 4); therefore, we pooled the data from the two systems. Significant differences in ambient noise were observed among different recording days; i.e., in *SPL_zp_*, *SPL_rms_*, *SEL_ss_* and *SEL_ws_* (Kruskal-Wallis χ^2^ = 27.18, df = 4, *p*<0.01; Kruskal-Wallis χ^2^ = 41.21, df = 4, *p*<0.01; Kruskal-Wallis χ^2^ = 41.21, df = 4, *p*<0.01 and Kruskal-Wallis χ^2^ = 215.34, df = 4, *p*<0.01; respectively, Table 4). In particular, significant variation was observed in *SPL_zp_* between SSP_22_ #38 and #41 vs #39 and #41 vs #36 (Duncan's multiple-comparison test; *p*<0.05) (Table 4). Significant differences were observed in *SPL_rms_*, *SEL_ss_* and *SEL_ws_* between SSP_22_ #39,#38 and #36 vs #32, between#39 and #38 vs #41 and #36 vs #41 (Duncan's multiple-comparison test; p<0.05) (Table 4). Significant ambient noise differences were observed between the morning (before pile driving) and afternoon (before extraction)of the same day; i.e., differences in *SPL_zp_*, *SPL_rms_*, *SEL_ws_* and *SEL_ws_* (Mann-Whitney U-test: z = −3.97, df = 214, *p*<0.05; Mann-Whitney U-test: z = −4.12, df = 214, *p*<0.05; Mann-Whitney U-test: z = −4.12, df = 214, *p*<0.05 and Mann-Whitney U-test: z = −4.66, df = 214, *p*<0.05; two-tailed; respectively) (Table 4).

**Table 3:** Descriptive statistics of the SPL_zp_, SPL_rms_ and SEL_ws_ values of the OCTA-KONG vibration.

	Data			Vibration			
			SPL_zp_ (dB re 1 µPa)	SPL_rms_(dB re 1 µPa)	SEL_ws_ (dB re 1 µPa^2^s)	N	Distance (m)
Piling	#32	Mean±SD	154.75±2.11	145.44±1.85	118.90±1.68	76	200
		Range	149.9–158.11	140.58–148.8	113.78–121.70		
	#39	Mean±SD	153.64±2.02	142.99±1.95	121.38±2.05	87	90–145
		Range	148.29–160.02	137.77–146.8	116.56–123.95		
	#38	Mean±SD	153.66±1.12	143.16±0.94	121.62±1.09	50	60
		Range	151.48–155.81	141.29–144.79	118.96–123.49		
	#41	Mean±SD	151.93±2.18	141.22±1.54	114.54±0.93	90	70
		Range	146.99–159.66	138.05–147.47	112.74–116.79		
	#36	Mean±SD	160.27±1.97	149.77±2.13	125.25±2.6	99	80
		Range	154.96–164.49	144.35–153.11	119.53–128.86		
Extract	#26a	Mean±SD	152.25±1.99	139.47±1.44	120.65±0.93	247	70
		Range	148.26–157.91	136.02–144.35	118.28–124.91		
	#26b	Mean±SD	151.27±4.98	137.7±5.6	123.17±5.47	1471	15–180
		Range	140.83–164	128.83–154.58	111.92–138.07		

Parameters are given as the mean ± standard deviation (SD), with the range denoting minimum and maximum values. SEL_ss_ was identical to SPL_rms_, N: sample size. Subscript 'a' denotes sound recorded by the BS recording system, and 'b' denotes sound recorded by the SM2M recording system.

**Table 4:** Descriptive statistics of the SPL_zp_, SPL_rms_ and SEL_ws_ of the ambient noise.

			SPL_zp_ (dB re 1 µPa)	SPL_rms_(dB re 1 µPa)	SEL_ws_ (dB re 1 µPa^2^s)	N
Piling	#32	Mean±SD	140.41±4.23^a^	124.72±3.51^abc^	99.12±2.6^abc^	53
		Range	131.24–147.66	117.36–133.31	94.47–103.90	
	#39	Mean±SD	142.45±3.49^bc^	126.22±2.45^bd^	108.34±2.63^bd^	67
		Range	135.94–148.28	121.58–133.52	103.56–112.68	
	#38	Mean±SD	140.2±3.09^b^	125.64±2.08^ce^	107.37±1.69^ce^	45
		Range	135.43–146.58	123.18–134.09	104.28–113.58	
	#41	Mean±SD	139.37±5.21^cd^	123.74±4.63^def^	100.71±1.98^def^	89
		Range	130.32–153.48	114.75–137.99	98.01–106.18	
	#36	Mean±SD	140.4±3.36^d^	127.34±2.9^af^	103.33±2.08^af^	54
		Range	134.15–151.71	122.17–134.01	101.16–114.17	
Extract	#26_a_	Mean±SD	142.07±3.39	129.3±3.09	102.57±1.58	94
		Range	134.42–153.1	121.77–139.16	99.95–106.94	
	#26_b_	Mean±SD	141.85±3.27	128.6±3.46	101.63±1.93	31
		Range	134.46–150.7	120.64–138.36	99.20–106.79	
	#26_c_	Mean±SD	142.12±3.31	129.1±3.14	102.34±1.62	125
		Range	134.42–153.1	120.64–139.16	99.20–106.94	

Parameters are given as the mean ±SD, with ranges denoting minimum and maximum values. SEL_ss_ was identical to SPL_rms_, Means with different lowercase superscripts refer to post hoc Duncan's multiple-comparison tests that yielded significant results (p<0.05) for OCTA-KONG pile driving. Subscript 'a' denotes sound recorded by the BS recording system, 'b' denotes sound recorded by the SM2M recording system and 'c' denotes the combined results of the BS and SM2M recording systems.

### Spectrogram, PSD and 1/3 octave band spectrum

**Figure 4:**
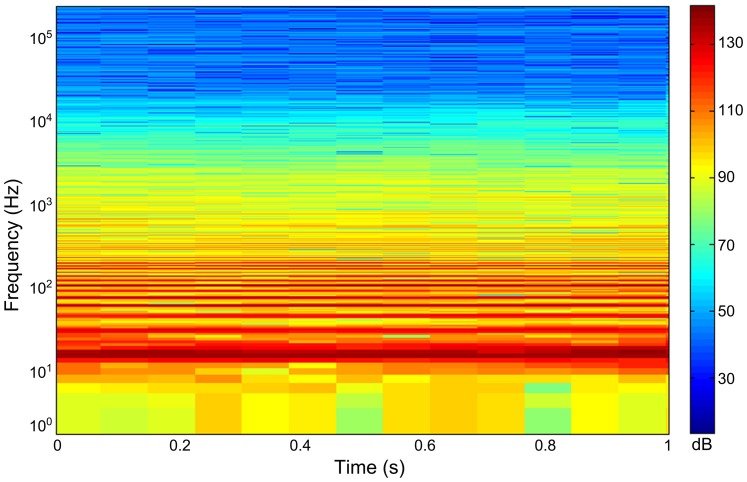
Spectrogram of the OCTA-KONG SSP_22_ #36 driving sound. Spectrogram configuration: temporal grid resolution, 76.80 ms; overlap samples per frame, 85%; frequency grid spacing, 1.95 Hz; window size, 262 144; FFT size, 262 144; window type, Hanning. The fundamental frequency of the vibration sound was 16 Hz.

**Figure 5:**
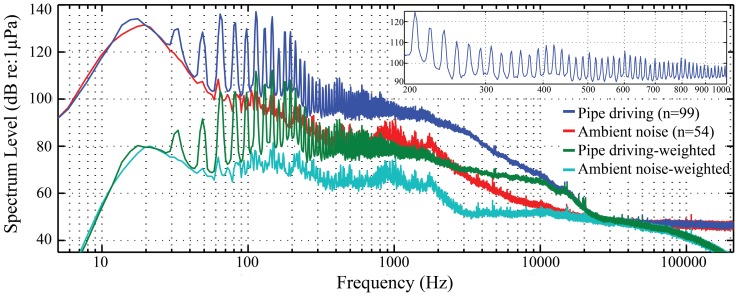
Power spectral density of the OCTA-KONG SSP_22_ #36 driving sound and noise. Spectrum configuration: temporal grid resolution, 76.80 ms; overlap samples per frame, 85%; frequency grid spacing, 1.95 Hz and normalized to 1 Hz; window size, 262 144; FFT size, 262 144; window type, Hanning. The inset in the upper right corner shows a magnified frequency scale of the unweighted piling sound. The fundamental frequency of the vibration sound was 16 Hz. Pile driving sounds were recorded at a distance of 80 m from the vibration hammer.

**Figure 6:**
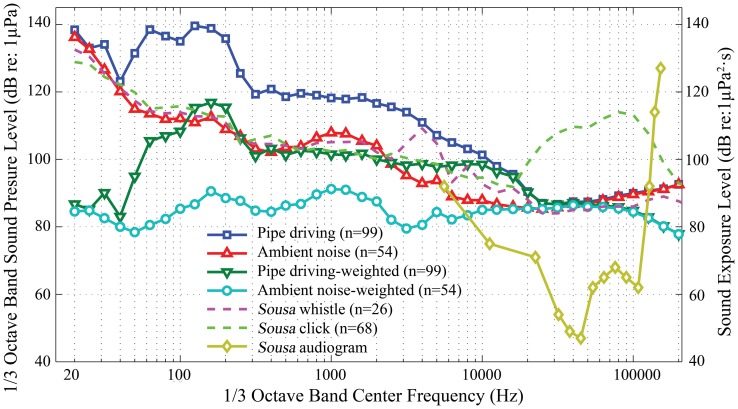
1/3 octave band frequency spectrum and A-weighted sound exposure level of the SSP_22_ #36 driving sound. Spectrum configuration: temporal grid resolution, 76.80 ms; overlap samples per frame, 85%; frequency grid spacing, 1.95 Hz; window size, 262 144; FFT size, 262 144; window type, Hanning. The *Sousa* audiogram was modified from previous sources [22], [23], with the lowest threshold at each frequency defining the merged audiogram curve. n denotes the number of samples. Pile driving sounds were recorded at a distance of 80 m from the vibration hammer.

The recorded fundamental frequency of the OCTA-KONG vibration ranged from 15 Hz (Fig. 3) to 16 Hz (Fig. 4, 5). The noise increments were below 20 kHz, with the dominant frequency and most energy contained below approximately 10 kHz (Fig. 4, 5, 6).

### SEL_ws_

The recorded *SEL_ws_* ranged from 112.74 dB to 128.86 dB and 111.92 dB to 138.07 dB for pile driving and pile extraction, respectively (Table 3).

### SL_zp_, SL_rms_, SSEL_ss_ and SSEL_ws_

**Figure 7:**
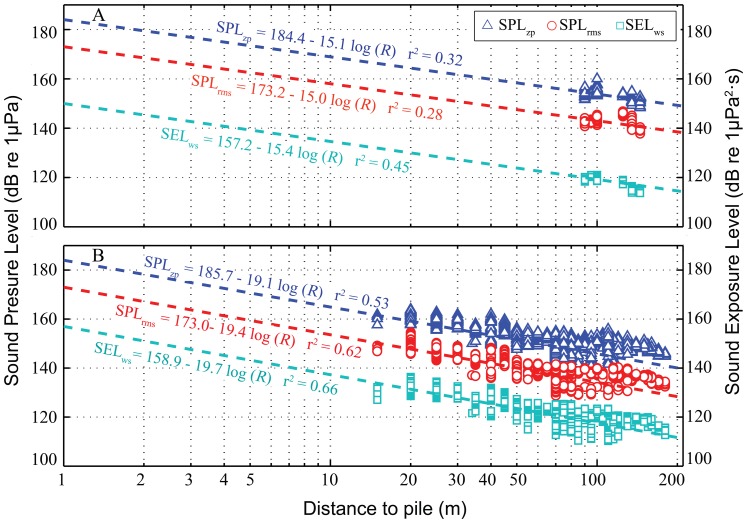
Broadband *SPL_zp_*, *SPL_rms_* and *SEL_ws_* of vibration sound as a function of distance from the noise source and the best-fit sound propagation model. A: OCTA-KONG SSP_22_ #39 driving; B: OCTA-KONGSSP_22_ #26 extraction. The sound propagation equations that predicted the received SPLs and SELs based on distance were derived by applying a least squares regression to the measurements obtained via the floating recording method for pile driving and extraction, respectively.

The best-fit sound propagation models for *SPL_zp_*, *SPL_rms_*, *SEL_ss_* and *SEL_ws_* for pile driving and pile extraction are shown in Figure 7A and 7B. The estimated mean *SL_zp_* during pile driving and extraction ranged from 179.79 dB to 189.01 dB and 185.70 dB to187.49 dB, respectively. The estimated mean *SL_rms_* and *SSEL_ss_* during pile driving and extraction ranged from 168.90 dB to 179.96 dB and 173.00 dB to175.26 dB, respectively. The estimated mean *SSEL_ws_* during pile driving and extraction ranged from 142.95 dB to 157.20 dB and 157.00 dB to158.90 dB, respectively (Table 5).

**Table 5:** *SL_zp_*, *SL_rms_* and *SSEL_ws_* of the OCTA-KONG pile driving and pile extraction and the audible range.

			OCTA-KONG	Ambient noise	Sensation level(dB)	Audible range(m)
Piling	SL_zp_	#32	189.5 (184.65–192.86)	140.41	49.09	1546
		#39	184.4	142.45	41.95	569
		#38	180.51 (178.33–182.66)	140.2	40.31	448
		#41	179.79 (174.85–187.52)	139.37	40.42	455
		#36	189.01 (183.7–193.23)	140.4	48.61	1456
	SL_rms_	#32	179.96 (175.1–183.32)*	124.72	55.24	3489
		#39	173.2	126.22	46.98	1212
		#38	169.83 (167.96–171.46)	125.64	44.19	818
		#41	168.9 (165.73–175.15)	123.74	45.16	939
		#36	178.32 (172.9–181.66)*	127.34	50.98	2068
	SSEL_ws_	#32	154.33(149.21–157.13)	99.12	55.21	2954
		#39	157.20	108.34	48.86	1324
		#38	149.00(146.34–150.87)	107.37	41.63	483
		#41	142.95(141.15–145.20)	100.70	42.25	527
		#36	154.55(148.83–158.16)	103.33	51.23	1802
Extract	SL_zp_	#26_a_	187.49 (183.5–193.15)	142	45.49	236
		#26_b_	185.7	141.85	43.85	196
	SL_rms_	#26_a_	175.26 (171.81–180.14)*	129.30	45.96	229
		#26_b_	173	128.6	44.40	192
	SSEL_ws_	#26_a_	157.00 (154.64–161.26)	102.57	54.43	557
		#26_b_	158.90	101.63	57.27	765

*SSEL_ss_* was identical to *SL_rms_*. The average levels of the OCTA-KONG and ambientnoise are provided. Numbers in parentheses indicate the range. Sensation level was derived by dividing the vibration sound by the ambient noise level. SLs and SSELs are re 1 µPa and 1 µPa^2^s, respectively. Subscript 'a' denotes sound recorded by the BS recording system, 'b' denotes sound recorded by the SM2M recording system. * denotes results that exceeded the proposed cetacean safety exposure level of 180 dB (*SPL_rms_*).

### Audibility range

The frequency-dependent sound absorption constant *a* was estimated at 0.0006 [35] for the specific pH of 8, a salinity of 33‰ and a water temperature of 20°C (measured at the piling sites during the sound recording period) at a frequency of 10 kHz; the majority of OCTA-KONG vibration noise power is found below this frequency (Figs. 4, 5, 6). The transmission loss equations with correlation to the distance *r* for *SL_zp_*, *SL_rms_*, *SSEL_ss_* and *SSEL_ws_* were 15.1 log_10_(*r*)+0.0006*r*, 15.0 log_10_(*r*)+0.0006*r*, 15.0 log_10_(*r*)+0.0006*r* and 15.4 log_10_(*r*)+0.0006*r*, respectively, for pile driving and 19.1 log_10_(*r*)+0.0006*r*, 19.4 log_10_(*r*)+0.0006*r*, 19.4 log_10_(*r*)+0.0006*r* and 19.7 log_10_(*r*)+0.0006*r*, respectively, for pile extraction. The estimated audible range of *SL_zp_* during pile driving and pile extraction ranged from 448 m to 1546 m and from 196 m to 236 m, respectively. The estimated audible range of *SL_rms_* and *SSEL_ss_* during pile driving and pile extraction ranged from 818 m to 3489 m and from 192 m to 229 m, respectively. The estimated audible range of *SSEL_ws_* during pile driving and pile extraction ranged from 483 m to 2954 m and from 557 m to 765 m, respectively (Table 5).

### Impact on *Sousa*

#### Auditory masking

The *Sousa* audiogram was revised from the two available audiograms [22], [23], with the lowest threshold at each frequency defining the merged audiogram curve. Both the OCTA-KONG vibration sound and the ambient noise level recorded in this study were above the threshold of the *Sousa* audiogram (Fig. 6). The 1/3 octave band sound pressure level of the *Sousa* click sound at a distance of less than 50 m with a dominant frequency range of 20 kHz to 200 kHz would not be masked by the OCTA-KONG vibration sound recorded at a distance of 200 m. However, the 1/3 octave band sound pressure level of the *Sousa* whistle recorded at a distance of less than 50 m with a dominant frequency range from 3 kHz to 6 kHz would be masked by the vibration sound recorded at a distance of 200 m (Fig. 6).

#### Cetacean safety exposure level

The maximum *SL_rms_* of SSP_22_ driving (#32 and #36) and extraction (#26) exceeded the established cetacean safety exposure *SPL_rms_* level of 180 dB (Table 5). However, the maximum *SL_rms_* of SSP_22_ pile driving of #38, #39 and #41 were lower than 180 dB (Table 5).

#### Physiological impact

All the calculated *SL_zp_* values of OCTA-KONG pile driving and pile extraction (with maximums of 193.23 dB and 193.15 dB, respectively) (Table 5) were well below 224 dB, the proposed *SL_zp_* threshold for the onset of TTS for mid-frequency cetaceans exposed to non-impulsive sound. However, the calculated *SSEL_cum_* values for pile driving of SSP_22_ #32, #39 and #36 and pile extraction of SSP_22_ #26 were 201.33 dB, 195.05 dB, 199.75 dB and 207.59 dB re 1 µPa^2^s, respectively (Table 6), exceeding the proposed 195 dB threshold for the onset of TTS in mid-frequency cetaceans exposed to non-impulsive sound. In addition, the calculated *SSEL_wcum_* for the pile driving of SSP_22_ #39 and pile extraction of SSP_22_ #26 was 179.05 dB and 191.41 dB re 1 µPa^2^s, respectively, greater than the proposed threshold of TTS onset at 178 dB. All the calculated SSELs were lower than the threshold of the onset PTS for mid-frequency cetaceans exposed to non-impulsive sound.

**Table 6:** SSEL_ss_, SSEL_ws_, SSEL_cum_ and SSEL_wcum_ of the OCTA-KONG vibration.

	Date	Sites	SSEL_ss_ (dB re 1 µPa^2^s)	SSEL_ws_ (dB re 1 µPa^2^s)	Duration(s)	10log(*t*)	SSEL_cum_ (dB re 1 Pa^2^s)	SSEL_wcum_ (dB re 1 µPa^2^s)
Piling	10/21/2013	#32	179.96	154.33	137	21.37	201.33*	175.70
	12/4/2013	#39	173.2	157.20	153	21.85	195.05*	179.05*
	12/13/2013	#38	169.83	149.00	142	21.52	191.35	170.52
	12/23/2013	#41	168.9	142.95	156	21.93	190.83	164.88
	1/4/2014	#36	178.32	154.55	139	21.43	199.75*	175.98
Extract	12/23/2013	#26	174.13	157.95	2219	33.46	207.59*	191.41*

Average SSEL_ss_ and SSEL_ws_ of #26 obtained from the results of the BS and SM2M recording systems (Table 5). *and ** denote results that exceeded the proposed acoustic threshold levels for the onset of TTS (178 dB and 195 dB for SSEL_wcum_ and SSEL_cum_, respectively) and PTS (198 dB and 215 dB for SSEL_wcum_ and SSEL_cum_, respectively), respectively.

## Discussion

Inshore marine mammals are highly susceptible to habitat loss, fragmentation, and degradation [5]. Marine mammals have a well-developed sense of hearing, and the importance of sound reception to these mammals makes them susceptible to the effects of anthropogenic noise [5].

The impacts of anthropogenic noise on marine life have been widely assessed [43]. The St. Lawrence River beluga (*Delphinapterus leticas*) may change its vocalization SPLs in direct response to changes in the noise field (Lombard effect) [44] or shift its frequency bands when exposed to vessel noise [45]. Killer whales (*Orcinus orca*) may adjust their vocal behavior, showing longer call durations [46], or exhibit a Lombard effect [47] to compensate for masking boat noise. Bowhead whales (*Balaena mysticetus*) may change migration routes and exhibit avoidance reactions when exposed to air gun noise [48] or travel at increased speeds in the presence of anthropogenic noise [5]. Harbor porpoises (*Phocoena phocoena*) tend to reduce their acoustic activity when exposed to the construction noise of pile driving [49] or reduce their buzzing activity when exposed to impulse noise from seismic surveys [50]. Bottlenose dolphins will significantly increase their whistle rate at the onset of an approach by a vessel [51], and Indo-Pacific bottlenose dolphins (*Tursiops aduncus*) tend to produce whistles with less frequency modulation at lower frequencies In habitats with greater ambient noise [52].

Acoustic impact models that estimate the effects of anthropogenic noise on the hearing and communication of fish and marine mammals by comparing noise spectra, audiograms and the vocalizations of the animal of interest have been widely applied, e.g., in research on the effects of ambient and boat noise on *Chromis chromis*, *Sciaena umbra* and *Gobius cruentatus* living in a marine protected area in Italy [53] and on the Lusitanian toadfish (*Halobatrachus didactylus*) in Portugal [54], on the impact of sounds resulting from construction and pipe-driving at an oil production island in Alaska on ringed seals (*Phoca hispida*) [34], on the potential effects of pile-driving at an offshore wind farm in the Moray Firth, NE Scotland on marine mammals [6], on the possible sensitivity of bottlenose dolphins to pile-driving noise [55], on the potential effects of underwater noise produced by whale-watching boats on killer whales in southern British Columbia and northwestern Washington State [56] and on the effects of the high-speed hydrofoil ferry in West Hong Kong waters on the Chinese white dolphin [57].

Transmission loss is correlated with bathymetry, substrate type, and sound speed profile along the direction of transmission [36], and the fit obtained for site-specific transmission loss may not apply to transmission in other directions from the source if these conditions are different in those directions [33]. Because the bathymetry and substrate type in the studied construction site are consistent, the site-specific empirical fit method that we used to determine the transmission loss can be applied to transmission in other locations.

### Spectrogram, PSD and 1/3 octave band frequency spectrum

The spectrograms and PSD levels allowed us to evaluate the detailed frequency composition of the signal (Figs. 4, 5); however, they did not consider the critical band theory of the mammalian auditory system. Therefore, they offer little insight into either how these mammals perceive noise or the extent of the masking effect of the noise PSD levels [9]. The 1/3 octave band sound pressure level information provided us with a starting point for evaluating the frequency components of the construction sounds that are audible to the dolphins [34]. Although there is little noise energy above the ambient noise levels between 20 kHz and 120 kHz, the Chinese white dolphin shows the greatest sensitivity to sound in this range, as is normal for toothed whales [5], and the majority of the noise increments above the ambient noise levels of 5.6 kHz to 20 kHz were greater than 15 dB (Fig. 6). Both the OCTA-KONG vibration noise and the ambient noise level were above the threshold of the *Sousa* audiogram at frequency bands between 5.6 kHz and 128 kHz (Fig. 6), indicating that sound detection in these frequency bands was limited by the ambient noise rather than by the Sousa audiogram.

### Impacts on *Sousa*

#### Sound masking

The dominant noise level of the OCTA-KONG operation was below 20 kHz, suggesting that *Sousa* clicks were not adversely affected (Fig. 6). This interpretation is further supported by the finding that the peak frequency of *Sousa* clicks ranges from 43.5 kHz to 142.1 kHz [23]. By contrast, the *Sousa* whistle, with a fundamental frequency ranging from 520 Hz to 33 kHz [15], was most susceptible to auditory masking and could be completely masked at a distance of 200 m (Fig. 6). As whistles play a significant role in dolphin communication, such auditory masking may disrupt activities such as feeding and sexual behavior [5]. The adopted safety zone of approximately 200 m radius, as suggested by NOAA [18], should be enlarged to a more conservative region of 500 m radius, as recommended by the Joint Nature Conservation Committee [58]. Beyond this distance, the audibility of certain OCTA-KONG vibrations to *Sousa* is negligible (Table 5).

#### Physiological impact

Although the *SL_zp_* of the SSP_22_ vibration was lower than the proposed physiological damage level, 60% (3 out of 5 piles) of the *SSEL_cum_* values during SSP_22_ driving, the *SSEL_wcum_* values during SSP_22_ #39 driving and both the *SSEL_cum_* and *SSEL_wcum_* values during SSP_22_ extraction exceeded the acoustic threshold levels for the onset of TTS (Table 6). In general, the *SSEL_cum_* and/or *SSEL_wcum_* values could exceed the PTS or TTS threshold in a multitude of ways, depending on the exposure levels and durations [25]. The average *SSEL_ss_* values for all six SSP_22_ sites were lower than the cetacean safety exposure level (180 dB) (Table 6); therefore, the surpassed *SSEL_cum_* and *SSEL_wcum_* levels were due to the prolonged duration of the operation (as a function of 10 log(*t*)). The average durations of OCTA-KONG vibration during pile driving and pile extraction are 3 min and 30 min, respectively, with a range of 2 min to 6 min and 20 min to 40 min, respectively (YP Wang, personal communication).

### Mitigation method

As the *SSEL_cum_* and *SSEL_wcum_* values were exceeded due to the prolonged sound exposure periods, the PTS could potentially be avoided by alternating the OCTA-KONG vibration with periods of inactivity, e.g., operations on nonconsecutive days to reduce the sound exposure. In addition, as several of the maximum *SL_rms_* values for OCTA-KONG vibration exceeded the cetacean safety exposure level, an air bubble curtain could be introduced. Such curtains can substantially reduce underwater noise at frequencies between 400 Hz and 6400 Hz [59]. During the present study, the power unit rotated primarily in the 1300 r/min–1500 r/min range; however, a maximum of 1700 r/min was used during the construction of the two artificial islands (YF Yang, personal communication),which may have introduced more intense operation noise. Moreover, in addition to the use of pings, “soft start” and “power down” techniques should be adopted [18]. Specifically, at the beginning of each pile installation or extraction, vibratory hammers should be activated at low power for 15 s, followed by a 1-min waiting period (i.e., at a duty cycle of 20%, repeated at least twice) before full power is achieved (i.e., a "soft start"). Additionally, if dolphins are observed within the exclusion zone during the in-situ vibration, operations should either cease or substantially reduce the vibration power (i.e., "power down"). Pile-driving operations should occur during periods when threatened or endangered species are less abundant, as suggested by NOAA [19].

### Limitations

The present study had two limitations: First, dolphin behavioral responses during pile driving and extraction were not addressed. In view of the limited current knowledge of the noise dose-response relationship, we are unable to assess whether the noise generated by the OCTA-KONG may cause behavioral disruption. Second, although the adopted audiogram was derived from two Chinese white dolphins of different ages [22], [23], there is individual variation in cetacean audiograms [60], [61]. Therefore, the two audiograms used here should not be considered representative of the hearing sensitivity of this species. In addition, the *Sousa* audiogram data were sparse and did not extend below a lower frequency limit of 5.6 kHz, further limiting noise exposure assessment at lower frequencies. Audiograms covering a wider frequency range for *Sousa* are needed to quantitatively analyze the impact of the noise.

## Conclusions and Future Research

The fundamental frequency of the OCTA-KONG vibration ranged from 15 Hz to 16 Hz, with noise increments below 20 kHz and a dominant frequency and energy below 10 kHz. The vibration zone detectable by *Sousa* extends beyond 3.5 km. *Sousa* clicks do not appear to be adversely affected, whereas *Sousa* whistles are susceptible to auditory masking; therefore, a safety zone of 500 m radius is proposed. Although the *SL_zp_* value of the OCTA-KONG was lower than the physiological damage level, the maximum *SL_rms_* value sometimes exceeded the cetacean safety exposure level, and the majority of *SSEL_cum_* and *SSEL_wcum_* values exceeded the acoustic threshold levels for the onset of TTS. Moreover, the TTS was due to the prolonged production of the vibration sound. These findings can help improve environmental impact analyses. Future research that evaluates the real-time noise conditions accompanying underwater construction and the associated behavioral responses of nearby dolphins is recommended to address, in a more direct and robust manner, the possible impacts of human-generated noise on these animals. An increased understanding of the dose effects of noise exposure will provide us with valuable information on how to mitigate possible impacts during the underwater project; this information is important for *Sousa* conservation. In addition, prey are a critical resource for cetaceans [62]–[64], but little is known about the effects of construction noise on fish [1], [65], [66]. Dolphins can identify and locate their prey through passive listening during the search phase of the foraging process [67], [68]. Therefore, further research is needed to identify the potential adverse impacts on fish, including the masking of prey sounds by anthropogenic noise, particularly of those species that are important prey for marine mammals.
